# Attentional networks in developmental dyscalculia

**DOI:** 10.1186/1744-9081-6-2

**Published:** 2010-01-07

**Authors:** Sarit Askenazi, Avishai Henik

**Affiliations:** 1 Department of Psychology and Zlotowski Center for Neuroscience Ben-Gurion University of the Negev, Beer-Sheva, Israel

## Abstract

**Background:**

Very little is known about attention deficits in developmental dyscalculia, hence, this study was designed to provide the missing information. We examined attention abilities of participants suffering from developmental dyscalculia using the attention networks test - interactions. This test was designed to examine three different attention networks--executive function, orienting and alerting--and the interactions between them.

**Methods:**

Fourteen university students that were diagnosed as suffering from developmental dyscalculia--intelligence and reading abilities in the normal range and no indication of attention-deficit hyperactivity disorder--and 14 matched controls were tested using the attention networks test - interactions. All participants were given preliminary tests to measure mathematical abilities, reading, attention and intelligence.

**Results:**

The results revealed deficits in the alerting network--a larger alerting effect--and in the executive function networks--a larger congruity effect in developmental dyscalculia participants. The interaction between the alerting and executive function networks was also modulated by group. In addition, developmental dyscalculia participants were slower to respond in the non-cued conditions.

**Conclusions:**

These results imply specific attentional deficits in pure developmental dyscalculia. Namely, those with developmental dyscalculia seem to be deficient in the executive function and alertness networks. They suffer from difficulty in recruiting attention, in addition to the deficits in numerical processing.

## Background

Developmental dyscalculia is generally defined as a disorder in mathematical abilities presumed to be due to a specific impairment in brain function [1,2]. Developmental dyscalculia is supposed to be a unique deficit that is not caused by a reading disorder (dyslexia), attentional disorder (ADHD/ADD- attention-deficit disorder) or general intelligence (IQ) problems. The present study aims to examine attention in developmental dyscalculia by employing a recently designed test of three attentional networks and their interactions [3].

### Characteristics of developmental dyscalculia: Does developmental dyscalculia involve domain-general or domain-specific deficits?

Children with developmental dyscalculia fail in a wide range of numerical tasks. For example, they present difficulties in retrieval of arithmetical facts [4-8], in using arithmetical procedures [e.g., [7]], and in solving arithmetical operations in general [9]. Recently, studies on developmental dyscalculia concentrated on basic numerical processing and found those with developmental dyscalculia exhibited an atypical effect; size congruency [10,11] magnitude comparisons [5,12] and subitizing [13].

Neuro-functional studies indicate that mathematical difficulties involve abnormalities in the structure or the activity of the parietal lobes, mostly the intraparietal sulcus. A focused infarct to the left intraparietal sulcus could produce primary acalculia [14]. Isaacs and co-workers [15] found reduction in gray matter in the left intraparietal sulcus in children born preterm who suffered from calculation deficits. A structural and functional abnormality in the right intraparietal sulcus was found in women with Turner syndrome who had dyscalculia [16]. Recent work by Price and co-workers [17] examined the activity in the brains of those with developmental dyscalculia and discovered reduced activity in the right intraparietal sulcus during non-symbolic magnitude processing. Finally, Cohen Kadosh and co-workers [18] showed that TMS (transcranial magnetic stimulation) to the right intraparietal sulcus in normal control participants induced a dyscalculia-like pattern. Accordingly, one line of thinking is that developmental dyscalculia is a domain-specific (pure) disorder that involves only deficits in basic numerical processing and is related to one biological marker (i.e., a deficit in the intraparietal sulcus) [e.g., see [19]].

Alternatively, some refer to deficits in arithmetic as a domain-general phenomenon. One of the main deficits in developmental dyscalculia is a difficulty in retrieval of arithmetical facts [e.g., see [4-8]]. It has been suggested that this difficulty is more related to deficits in attention, working memory or long-term memory than to deficits in conceptual knowledge of arithmetic [e.g., see [20]]. In addition, there are indications that mathematical abilities are directly related to general abilities such as executive functions [e.g., [21]] and verbal or visuo-spatial working memory [e.g., [22]]. Finally, there are indications that the developmental course of the numerical distance effect is domain-general rather than domain-specific [see [23]]. Moreover, it has been suggested that mathematical learning difficulties are characterized by heterogeneity in symptoms and possibly in deficient mechanisms [24].

Karmiloff-Smith [25] suggested that developmental disorders characterized by a domain-specific end state can stem from a domain-general starting point. In addition, she suggested that neuropsychological dissociation studies of brain injuries and stroke patients that have dramatically influenced cognitive research may not apply to developmental disorders such as developmental dyscalculia. First, the basis of developmental disorders is believed to be genetic and one could not expect a one-to-one correlation between genes and specific cognitive functions (such as a deficit in processing of quantities in developmental dyscalculia). Second, there are compensation mechanisms that operate throughout development and change the observed deficits during adolescence. Third, some of the tests employed in the screening process of research participants (e.g., screening for attentional deficits in developmental dyscalculia) are not sensitive enough to reveal deficits in the "preserved" domain. Hence, it is possible that an abnormal expression of genes affects multiple aspects of development with the strongest effect being on one specific aspect. In the case of developmental dyscalculia, this could be core numerical processing.

The present study investigates attention, in a group of those with "pure" developmental dyscalculia. Namely, the participants have no indication of deficits in commonly used tests of attention and reading, and have a normal level of intelligence. We focus on attention because the intraparietal sulcus, involved in number processing and possibly which is abnormal in those with developmental dyscalculia, has a critical role in orienting of attention [see [26]]. Moreover, several researchers have suggested that some of the difficulties in developmental dyscalculia may be related to attention [e.g., see [20]]. The next section discusses possible abnormalities in attention in the developmental dyscalculia population.

### Attention and developmental dyscalculia

Shalev and co-workers [27] found that children diagnosed as having developmental dyscalculia had a higher mean score on an attentional problem subscale than matched controls. A similar pattern of results was found in Lindsay et al.'s [28] study--the developmental dyscalculia group in their study presented more commission and omission errors compared to the controls in the Conners' Computerized Continuous Performance Test (CPT). In addition, many deficits that characterize developmental dyscalculia can be connected to deficits in recruiting attention.

Rubinsten and Henik [10,11] examined developmental dyscalculia participants using the numerical Stroop task and discovered a lack of facilitation. They concluded that the ability to connect Arabic numerals to internal magnitudes is damaged in those with developmental dyscalculia. However, deficits in the executive functions network in the developmental dyscalculia population can also influence performance in the numerical Stroop task. In Stroop and Stroop-like tasks, a multi-dimensional object is presented and participants have to attend to one dimension while ignoring other dimensions. Performance in these tasks is considered to be based, among other things, on selective attention abilities and on executive functions, examined frequently in conflict situations [e.g., [29,30]]. Moreover, the anterior cingulate cortex is considered to be involved in conflict monitoring [e.g., [31]]. In a study by Kaufmann et al. [32], activity in the anterior cingulate cortex was discovered during the numerical Stroop task. In addition, we recently found that normal participants presented a developmental dyscalculia-like pattern in the numerical Stroop task under a condition of attentional load, namely, they showed a lack of facilitation [33].

Those with developmental dyscalculia have a smaller subitizing range [13]. Subitizing is a fast and accurate evaluation of a small set of objects [13]. However, it was recently suggested that subitizing may be modulated by attention. A recent study by Railo and co-workers [34] examined the role of attention in the subitizing process and discovered that when attention is limited, the subitizing range decreases to 2 dots. In addition, attentional training increases the subitizing range [35].

Several studies directly proposed that those with developmental dyscalculia suffer from deficits in executive functions [e.g., [36,37]] or in working memory [e.g., [13,21,38]]. In contrast, Censabella and Noël [39] found no evidence for deficient executive functioning in mathematically disabled (MD) children. They used the Stroop and the flanker tasks to examine the inhibition ability in MD children and matched controls. The ability to inhibit irrelevant information is considered to be part of the executive function network. Their results indicated that MD children showed normal performance on these tasks. No group differences were found in their study.

Some of the studies that were described above did not differentiate between pure developmental dyscalculia and the co-morbidity between developmental dyscalculia and ADHD [e.g., [13,28,36,38,40]], thus it is not clear whether the attentional difficulties in those with developmental dyscalculia are part and parcel of their dyscalculia or of the co-morbid deficit (i.e., ADHD). To this end, it is important to exclude participants with co-morbidity between ADHD and developmental dyscalculia [e.g., [10,11]]. The present study explores attentional deficits in developmental dyscalculia by studying developmental dyscalculia participants not suffering from ADHD.

### Three brain networks of attention

Early discussions defined attention as a cognitive process that selectively concentrates on one aspect of the environment while ignoring other aspects. This early definition viewed attention as one system. More recent works differentiated between several networks of attention. For example, Posner and Petersen [41] and later Posner and others [42-46] defined three separate networks of attention in the brain, which differ from one another in brain locations and functions. These networks carry out the functions of alerting, orienting, and executive control.

The alerting network is related to the awakeness state. Its role is to activate and preserve attention. Brain tissue involved in this network includes frontal and parietal regions of the right hemisphere. The alerting network is based on the distribution of the brain norepinephrine system [47,48].

The orienting network is involved in moving attention to a specific location in space. Attention can be shifted by moving the eyes, head or body position or without changing position [49]. The function of the orienting network can be stimulus-driven (exogenous, automatic, or bottom-up) and goal-directed (endogenous, voluntary, or top-down). The orienting network involves the superior parietal lobes, in particular, the intraparietal sulcus [26]. The parietal lobes are involved in suppression of old attended locations and in voluntary movement of attention to new locations [e.g., see [50,51]]. In addition, other brain areas are considered to be involved in the orienting system, that is, the superior colliculus [52] and the thalamus [53].

The executive control of attention is the third system and is involved in conflict situations. Commonly, the Stroop and the flanker tasks are employed to study this system. The frontal lobe, mostly the midline frontal areas (anterior cingulate cortex) and the lateral prefrontal cortex [30,54] subserve the executive system. It has been suggested that the midline areas (i.e., anterior cingulate cortex) are responsible for conflict monitoring and the lateral areas (i.e., lateral prefrontal cortex) are responsible for inhibition of irrelevant responses and maintaining task requirements [55,56].

In 2002, Fan et al. created a general test for the three attention systems--the Attention Network Test (ANT). The basic assumption of the ANT is that the three attentional networks are isolable. In contrast with this claim, Callejas et al. [3] reported an interaction between the three attentional networks and created a new test, the ANT-I, which examines the three systems and their interactions (see appendix 1). Several populations have already been tested with the ANT (e.g., children [57], borderline disorder [58]; ADHD [59]).

Rueda et al. [57] examined children aged 6 to 10 years old and compared their performance in the 3 attentional networks. Alertness was fully developed by the age of 10, the executive functions network was fully developed by the age of 7, and the orienting network was found not to be modulated by age.

Booth et al. [59] examined participants with ADHD using the ANT test. They reported that only the alertness network of ADHD participants presented an abnormal pattern of performance. In addition, they revealed that subtypes of ADHD presented different patterns of abnormal performances: the ADHD of the combined subtype (ADHD/C) showed a smaller alerting effect compared to typically developing children, whereas the inattentive subtype (ADHD/IA) showed a larger alerting effect compared to typically developing children, that is, a greater benefit from the high alerting state compared to controls.

### The present study

Very little is known about attention deficits in developmental dyscalculia, hence, this study was designed to provide the missing information. Most of the studies see developmental dyscalculia as a pure disorder that does not involve deficits in attention. In the present study we would like to suggest that even in "pure" developmental dyscalculia one can observe abnormalities in attention. We examined attention abilities of participants suffering from developmental dyscalculia using the ANT-I test [3].

The ANT-I test examines executive function by using a variation of the flanker task [60]. In the flanker task the participants have to attend to one object while ignoring other objects. In the version that was used in the present study, participants were asked to respond to a central target and ignore flanking distractors. It is hard to determine whether this network would be damaged in developmental dyscalculia participants because the evidence provided by the literature is inconsistent. Orienting of attention is tested by using spatial cueing. Based on earlier findings [e.g., [15-17]], we hypothesized that developmental dyscalculia participants would present an abnormal pattern of attentional orienting. Alertness was tested by the presentation of a tone at the beginning of a trial. The intraparietal sulcus is considered to be involved both in the orienting network and in the alertness network. Due to the anatomical proximity of the alertness network and the orienting network, similar to our prediction about the orienting system, we hypothesized that developmental dyscalculia participants would present abnormal alerting as well.

## Methods

### Participants

Twenty-eight students from Ben-Gurion University of the Negev and Achva Academic College participated in the experiment. Fourteen of them were diagnosed as suffering from developmental dyscalculia and the other fourteen were age and sex matched controls. The controls did not have any learning or other disabilities. All students were paid 20 NIS ($5) for participation in the experiment or were given a course credit.

#### Control group

The control group consisted of 14 participants, of which 11 were female, mean age 24.3 years (*SD *= 1.8). All of them were age and sex matched to the developmental dyscalculia group. None of the participants in this group were diagnosed as having developmental dyscalculia. All of them took the arithmetic, reading, Raven's Progressive Matrices, and CPT II tests and did not show any learning disability.

#### Developmental dyscalculia group

The developmental dyscalculia group was composed of 14 participants, of which 11 were female, mean age 23.9 years (*SD *= 1.8). All the participants in the group were diagnosed as having developmental dyscalculia according to Rubinsten and Henik's [10,11] criteria.

All of them were college students that volunteered for the experiment due to severe difficulties in numerical processing, with no indication of deficits in reading or attention. Approximately 50 participants that met these criteria volunteered for the experiment. Out of these 50, thirty passed the interview stage and underwent a full diagnosis. Eighteen participants met the developmental dyscalculia criteria. Four were excluded from the developmental dyscalculia sample; two due to being diagnosed as suffering from ADHD and two due to relatively low IQ.

Before the beginning of the experiment, every candidate was tested individually for developmental dyscalculia, dyslexia, IQ and ADHD. For the dyscalculia assessment, we develop a new mathematical ability test (see below) based on the assumptions suggested by Rubinsten and Henik. A participant that scored two standard deviations below the score of the norm (that was based on the scores of 40 students) was diagnosed as suffering from developmental dyscalculia. Performance was measured by reaction time (RT) and accuracy.

### Preliminary testing - mathematical ability test

The mathematical ability test was administered individually. Time was measured for every sub-test and was divided by the number of trials in the sub-test (when applicable) to give an average RT. The test was divided into two parts, the first part dealing with number comprehension and production, and the second part dealing with calculation.

#### Part 1 - number comprehension and production

The sub-tests in Part 1 were as follows.

1. *Comparing digits*. Comparison of 8 pairs of numbers: two pairs of three-digit numbers, 3 pairs of four-digit numbers and 3 pairs of five-digit numbers. The participants had to mark one of the following signs between the numbers: smaller than (<), larger than (>) or equal (=) (e.g., 987_ 432, mark <, > or =; the correct answer was: 987 > 432).

2. *Counting*. The participant had to count forward 4 times (e.g., count from 230-245) and backward 4 times (e.g., count from 245-230). The initial number was a three- or four-digit number.

3. *Estimation of quantity*. The participants had to estimate the result of an operation between two numbers. They were instructed not to calculate the exact result. They had to give the answer as soon as possible, with no time restriction. For eight of the estimations (2 additions, 2 subtractions, 2 multiplications, 2 divisions) the participants had to decide between 2 choices (e.g., Is 61/10 larger or smaller than 10?), while, the other eight estimations were standard format questions (2 additions, 2 subtractions, 2 multiplications, 2 divisions--e.g., what is the estimated result for 298 × 190; a good estimate would be 60,000).

4. *Series progression (non-numerical)*. The participants had to answer some ordinal questions, for example, name the days of the week, name the months in the year, etc.

5. *Numerical series*. The participants had to complete 14 arithmetical series, 3 of them contained three-digit numbers and the rest, two-digit numbers (e.g., 20, 40, 60, _____, _____, _____; the correct answer:, 80, 100, 120).

6. *Comparing fractions*. The participants had to compare 6 pairs of fractions and 14 pairs of decimals. They had to choose one of the symbols representing smaller than (<), larger than (>) or equal (=), to describe the relationship between two members of a pair (e.g., 1/4 _ 1/2, mark <, > or =; the correct answer was 1/4 < 1/2).

7. *Verbal problems*. Nine verbal problems were presented and participants were asked to: 1) Circle the correct operation for the given problem (addition, subtraction, multiplication or division). 2) Write down the representative equation. 3) Solve the equation. Take for example, the verbal problem: In a school, there are 40 students in a class. There are 7 classes in this school. How many students are studying in this school? Answer: step 1 - the correct operation is multiplication; step 2 - the correct equation is: 40 × 7 = ; and step 3 - the correct answer is: 280.

#### Part 2 - calculation

There were 6 sub-tests in Part 2 as follows.

1. *Simple pure operations*. Single-digit operations were administered (9 additions, 9 subtractions, 9 divisions and 9 multiplications). Each kind of operation appeared separately.

2. *Simple mixed operations*. Single-digit operations were administered (5 additions, 5 subtractions, 5 divisions and 5 multiplications), mixed in one block in a random order. The equations had the same level of difficulty as in the pure block.

3. *Horizontal operations*. Two- or three-digit numbers were presented in a horizontal alignment (8 additions, 8 subtractions, 8 divisions and 8 multiplications), for example, 554 + 96 =.

4. *Vertical operations*. Two- or three-digit numbers were presented in a vertical alignment (8 additions, 8 subtractions, 8 divisions and 8 multiplications), for example,

554

+ 96

5. *Decimals*. Decimal equations were administered (4 additions, 4 subtractions, 4 divisions and 4 multiplications), for example, 0.5 + 0.96 =.

6. *Fractions*. Fraction equations were administered (10 additions, 10 subtractions, 6 divisions and 7 multiplications), for example, 1/4 + 1/2 =.

### Results of preliminary testing

The results of the developmental dyscalculia and control participants in the mathematical ability test are presented in Tables 1 and 2. The RTs in the tables are for the various sub-tests. In most subtests it took developmental dyscalculia participants longer to respond than the control participants, both in number comprehension and production (particularly in comparing digits, counting, series, and fraction comparison), and in calculation (particularly in simple operations, horizontal operations and fractions). In contrast to the wide-scale differences that were found between the groups in the RTs measurements, a higher error rate for the developmental dyscalculia group compared to controls was observed in the following sub-tests: number comprehension and production (particularly in counting and series), and in calculations (particularly in horizontal operations--division; vertical operations--adding, subtraction; decimals--subtraction, multiplication; fractions--subtraction).

**Table 1:** Arithmetic battery part 1: number comprehension and production - mean reaction time and mean error rate percentage

Subtest	Control	DD
Comparing digits	14 sec (0%)	20 sec** (7%)

Counting	108 sec (0.3%)	125 sec** (37.5%)**

Series progression (non-numerical)	17 sec* (25%)	14 sec (43%)

Series	88 sec (1%)	119 sec** (6%)

Comparing fractions	42 sec (0%)	68 sec** (7%)**

Verbal operation	281 sec (11%)	308 sec (23%)

*Note*. An asterisk indicates a significant difference between the performances of the developmental dyscalculia group and the control group. * *p *< 0.05, ** *p *< 0.01.

**Table 2:** Arithmetic battery part 2: calculation - mean reaction time and mean error rate percentage

	Control	DD
**Simple operations (RT)**	61 sec	123 sec**

Addition	1.35%	4%

Subtraction	0%	5%

Multiplication	2.5%	4.5%

Division	5%	8.5%

**Horizontal operations (RT)**	13 sec	21 sec**

Addition	6%	17.5%

Subtraction	23%	23%

Multiplication	6%	15%

Division	2%	17%*

**Vertical operations (RT)**	625 sec	822 sec

Addition	5%	18%**

Subtraction	13%	32%**

Multiplication	26%	23%

Division	25%	42%

**Decimals (RT)**	238 sec	294 sec

Addition	12%	16%

Subtraction	28%	58%**

Multiplication	31%	70%*

**Fraction (RT)**	547 sec	658 sec*

Addition	13%	28%

Subtraction	9%	25%*

Multiplication	9%	21%

Division	5%	14%

*Note*. An asterisk indicates a significant difference between the performances of the developmental dyscalculia group and the control group. * *p *< 0.05, ** *p *< 0.01.

For reading assessment, we used a reading test that was composed and published by Shalev and colleagues [2] and standardized in a separate study [8]. Our sample of developmental dyscalculia students did not have any reading difficulties and there were no differences in the scores of any reading tests compared with the control group (for more details see Table 3). We converted participants' scores on the Raven's Progressive Matrices to IQ scores. All the participants achieved an average or above average IQ score.

**Table 3:** Preliminary test score error rates (standard deviation in parentheses)

Subtest-attention and reading	Control	DD
Omission	2.21 (4.45)	1.33 (2.16)

Commission	8.25 (5.3)	12.67(4.9)

Chance for ADHD	28% (16.6)	27% (14.7)

Reading test (number of error)	10.9 (7.2)	9.6 (7)

*Note*. The two groups were not significantly different on any measure.

For attention deficits examination, we used the Conners' Continuous Performance Test II (CPT II V.5). In this test, a single letter is presented at the center of the screen. The participants are asked to press the right mouse key when they spot a letter in the alphabet (e.g., A, B, C), except for the letter 'X'. If the letter 'X' is presented, they have to withhold their response until the letter disappears. In addition, in a questionnaire we asked our participants if they thought they had a possible deficit in attention. We excluded two participants from the developmental dyscalculia group due to deficits in attention in the CPT and in the questionnaire. There were no significant differences in attention abilities between the developmental dyscalculia and the control group. Mean chance for ADHD was 27% in the developmental dyscalculia group and 28% in the control group *[F < 1]*. In addition, there was no significant difference between the control and the developmental dyscalculia group in the omission *[F < 1] *or commission rates *[ns] *(for more details see Table 3).

### Stimuli and design

#### ANT-I

The ANT-I was administered according to Callejas et al. [3]. Briefly, the procedure was a combined cuing and flanker task. In each trial a line of 5 arrows was presented in the middle of the screen. The participants were instructed to attend to the middle arrow and to decide whether it was pointing to the left or to the right. Two arrows flanked the target arrow on either side and could appear in the same direction as the target arrow (congruent condition e.g. ← ← ← ← ←) or in the opposite direction (incongruent condition e.g. ← ← → ← ←). The set of arrows (target and flankers) could appear above or below fixation. An asterisk was used as a location cue and could appear in the same location as the target (valid condition), the opposite location (invalid condition), or not appear at all (non-cued condition). Alertness was manipulated by the presence or absence of a short duration, high frequency tone. The participants had to press the left-hand key if the central arrow was pointing left and a right-hand key if it pointed right.

### Procedure

In each trial, participants were presented with a fixation point of a variable duration ranging from 400 to 1,600 ms. In half of the trials, fixation was followed by a 2,000 Hz 50 ms tone. After a stimulus onset asynchrony (SOA) of 400 ms, a cue was presented for 50 ms in 2/3 of the trials. Half of the time the cue was presented at the location of the target (valid trials) and the other half, at the location opposite to that of the target (invalid trials). Fifty ms after this, the target was presented until a response.

## Results

Error rates were generally low [1% in the developmental dyscalculia group (*SD *= 2.25) and 1% in the control group (*SD *= 3.05)] and therefore were not analyzed. For every participant in each condition, mean RT was calculated (only for correct trials). These mean RTs were subjected to a four-way analysis of variance (ANOVA) with group as the only between-subject factor and congruity (congruent vs. incongruent), alertness (no-tone vs. tone) and cueing (invalid, non-cued, valid) as within-subject factors.

Three main effects were significant. Responses were faster in congruent trials (mean RT = 566 ms) than in incongruent trials (mean RT = 656 ms) [*F*(1, 26) = 189.4, *MSE *= 3,593, *p *< 0.001]; in trials with a tone (mean RT = 597 ms) than trials with no tone (mean RT = 624.7 ms) [*F*(1, 26) = 55.6, *MSE *= 1,132, *p *< 0.01]; and for valid compared with non-cued and invalid trials (640 ms for invalid trials, 610 ms in non-cued trials and 583 ms for valid trials) [*F*(2, 52) = 69.5, *MSE *= 1,340, *p *< 0.01]. As predicted by Callejas et al [3], the interaction between alertness and congruency was significant [*F*(1, 26) = 27.7, *MSE *= 269, *p *< 0.001]--the congruity effect was larger in the tone trials than in the no-tone trials. A significant interaction was also found between cueing and congruency [*F*(2, 52) = 11.9, *MSE *= 452, *p *< 0.001]. The interaction between alertness and cueing was also significant [*F*(2, 52) = 16.9, *MSE *= 759, *p *< 0.001].

**Figure 1:**
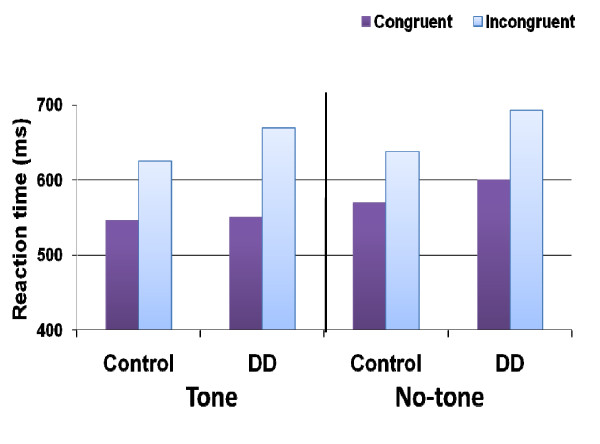
**RTs as a function of group, alertness and congruity**.

There was no main effect of group but the interaction between group and congruity was significant [*F*(1, 26) = 5.9, *MSE *= 3,593, *p *< 0.05]. The congruity effect (that examined the executive function network) was larger in the developmental dyscalculia group compared to the control group. The interaction between group and alertness was also significant [*F*(1, 26) = 6.6, *MSE *= 1,132, *p *< 0.05]--the tone effect was larger in the developmental dyscalculia group compared to the control group. The interaction between congruency and group was moderated by alertness [*F*(1, 26) = 4.8, *MSE *= 277, *p *< 0.05]. In the no-tone condition, the difference in the congruity effect between the two groups was marginally significant [*F*(1, 26) = 3.4, *MSE *= 1,725, *p *= 0.08], whereas in the tone condition, the difference in the congruity effect between the two groups was significant [*F*(1, 26) = 7.73, *MSE *= 1,725, *p *< 0.01]. That is, the congruity effect of the developmental dyscalculia group was larger than that of the control group regardless of alertness but the difference between the two groups was larger in the tone condition compared to the no-tone condition (see Figure 1).

**Figure 2:**
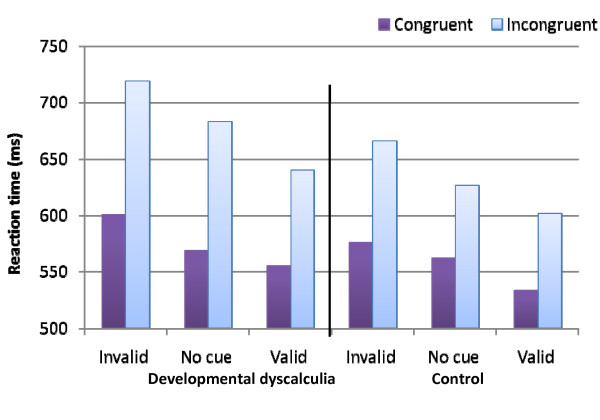
**RTs as a function of group, cueing and congruity**.

The interaction between congruity and cueing (that examined the orienting network) was moderated by group [*F*(2, 52) = 4.4, *MSE *= 452, *p *< 0.05] (see Figure 2). Similar to a previous report [3], the control group presented an interaction between congruity and cueing. The congruity effect was larger in the invalid trials compared to the non-cued and the valid trials [*F*(1, 13) = 6.3, *MSE *= 831.5 , *p *< 0.05]. In particular, the congruity effect was similar in the non-cued and the valid trials [*F *< 1], and it was smaller than the congruity effect presented in the invalid condition [*F*(1, 13) = 63.3, *MSE *= 813, *p *< 0.05]. In contrast, the developmental dyscalculia group presented a different pattern; the congruity effect was similar in the non-cued and the invalid trials [*F *< 1], and it was smallest in the valid condition compared to the non-cued and the invalid conditions [*F*(1, 13) = 21.9, *MSE *= 417, *p *< 0.01]. The basis of this triple interaction was the group difference in the size of the congruity effect of the non-cued condition (larger in the developmental dyscalculia group compared to the controls). The difference between the invalid and the valid trials (without the non-cued condition) was not modulated by the group factor [*F *< 1]. In addition, the interaction between cue (invalid vs. valid) and congruity was not modulated by group [*F *< 1], that is, when the general analysis included only two validity conditions (valid and invalid), the interaction between group, validity and congruity was not significant. This indicates that the developmental dyscalculia and control groups had a similar congruency effect in the invalid and valid conditions.

## Discussion

Let us summarize the main results. 1) The control group replicated the results found in Callejas et al.'s [3] study, that is, they presented all three main effects--congruity, alerting, and cueing. In addition, the congruity effect was larger under high alertness than low alertness, congruity was also modulated by cueing, and it was largest in the invalid condition. 2) The developmental dyscalculia group did not differ from the control group in general RT or error rates. However, the developmental dyscalculia group presented a congruity effect and an alertness effect that were larger than the effects presented by the controls. 3) The difference in the congruity effect between the two groups was larger in the tone condition in comparison to the no-tone condition. 4) The triple interaction between congruity, orienting and group was significant due to the larger congruity effect presented by the developmental dyscalculia group in the no cue condition. The two groups showed comparable congruity effects in the valid and invalid conditions. Note that regardless of the validity condition, the developmental dyscalculia group presented a larger congruity effect than the controls did. Developmental dyscalculia participants presented a unique attention profile in two attentional networks--the executive and the alerting networks. In what follows, we discuss this pattern of results.

### Executive functions

Discussion in the literature suggests that the executive function network is needed when an individual is requested to make a decision, for conflict resolution, error monitoring, planning an action, and inhibiting a response. The present study reveals a deficit in the ability to inhibit responding among developmental dyscalculia participants or a deficit in conflict resolution.

The connection between mathematical abilities and inhibition of response or executive functions was previously discussed [21]. Bull and Scerift examined executive functions in 3^rd ^graders and found a strong correlation between their mathematical abilities and their executive functions. It has been reported that children of lower mathematical ability do indeed show difficulties on tasks that measure the ability to inhibit both prepotent information (Stroop interference) and learned strategies (Wisconsin card sorting task, preservative responses). In relation to developmental dyscalculia, difficulties in executive functions were reported earlier [e.g., [36,37]]. A recent neuro-anatomical study by Rotzer and colleagues [61] adds to the behavioral results. They discovered abnormalities in gray matter volume of their developmental dyscalculia participants compared to controls in frontal sites that are believed to be a part of the executive network. The gray matter volume of developmental dyscalculia participants was smaller in the bilateral middle frontal gyrus, the left inferior frontal gyrus, the bilateral anterior cingulum and the right intraparietal sulcus.

How can the deficit in the executive function network be related to the arithmetic difficulties in developmental dyscalculia? First, it is well known that developmental dyscalculia participants present deficits in retrieval of arithmetical facts [4-8]. The deficit in retrieval of arithmetical facts is related to difficulty in conflict resolution [4-8]. When children acquire knowledge of arithmetical expressions (e.g., 3 + 5), they have to reject various alternative solutions and to associate the expression with the correct solution (e.g., 8) [62]. This could be especially true when children learn the various operations, so that solutions associated with various operations might pop-out automatically [i.e., the associative confusion effect, [63]]. Difficulty in conflict resolution could produce deficits in retrieval of the correct solution. It was reported that the executive function network was activated more during passive viewing of incorrect solutions (e.g., 2 + 2 = 5) compared to correct solutions (e.g., 2 + 2 = 4) [64]. Moreover, Thompson-Schill, and co-workers [65] suggested that the left inferior frontal gyrus is involved in the retrieval of facts from semantic memory and the differentiation between correct and incorrect facts. Abnormalities of these frontal structures in those with developmental dyscalculia might be responsible for this deficit [62]. In addition, this same frontal structure and also the anterior cingulate gyrus may also be responsible for the size congruity deficit reported in developmental dyscalculia [10,11].

In contrast to previous results and the current results, a recent study by Censabella and Noël [39] suggested that children with MD and controls have comparable executive functions. As in the present study, Censabella and Noël used the flanker task to examine the executive network. However, there were some differences between their investigation and the current study. First, the present study did not examine the flanker task only; the influence of cueing and alertness conditions was also monitored. It is possible that manipulation of several variables at the same time, and possibly other characteristics of our study, created a situation with more cognitive load, which was harder for the developmental dyscalculia participants to cope with. Second, it is also possible that age had a major effect. The executive function network develops until the age of 19 years old [66]. Huiziga and colleagues found that in a flanker task there was a larger congruity effect for a group of 7 year olds compared to 11 year olds and a larger congruity effect for a group of 11 year olds compared to 15 year olds. It is possible that a not-fully-developed executive network prevented the appearance of group differences in the Censabella and Noël study.

### Comparison of executive networks in developmental dyscalculia and ADHD

The present work found developmental dyscalculia participants to have a deficient executive network, whereas a former study that used a similar test (ANT) found no such deficit in children with ADHD [59]. However, other studies of people with ADHD showed they presented difficulties in the executive network [67-70]. One of these reports employed the go-no-go task. In this task, participants are instructed to respond to the 'go' signal (e.g., as in the CPT II task, all the alphabet letters except the letter 'X', see Method for more details regarding the Connors' attention examination) and to ignore the 'no-go' signal (e.g., as in the CPT II task, the letter 'X'). The flanker task and the go-no-go task share the same requirement to inhibit a response, and the current study suggests that this ability was deficient in the developmental dyscalculia group. The flanker task is also used to study conflict situations and it was found to activate the anterior cingulate cortex. Hence, it is possible that those with developmental dyscalculia have difficulty in either inhibition of irrelevant responses or in monitoring conflict situations or both.

As mention earlier, developmental dyscalculia participants presented abnormalities in the gray matter volume of frontal areas [61]. Abnormalities in the gray matter volume were found in those with ADHD in the right putamen/globus pallidus regions [71]. Due to connections between these areas and the frontal lobe, such a deficiency may modulate activity of the frontal cortex.

To sum up, the neuroimaging studies of those with developmental dyscalculia and ADHD demonstrated abnormalities in areas of the frontal lobe or areas connected to the frontal lobes. In line with this finding, both the ADHD and developmental dyscalculia groups seem to suffer from a deficit in the executive network.

### Deficit in alerting in developmental dyscalculia

The larger alerting effect in the developmental dyscalculia group found in the current study is similar to the results that were previously found in populations that have deficits in attention. For example, this was found in ADHD children of the inattentive subtype, but not in those of the combined subtype [59]. In addition, senior and Alzheimer's disease patients presented a larger alerting effect compared to younger controls [72]. Deficiencies in alertness were found in brain injuries of the posterior cortex [73]. This fits in with the suggestion that alerting involves the intraparietal sulcus [74]. As discussed in the introduction, the intraparietal sulcus is involved in numerical processing and in particular, in the size congruity task [e.g., [18]]. It is possible that an intraparietal sulcus abnormality in students suffering from developmental dyscalculia is responsible for their alerting deficit.

Posner and Raichle [75] suggested that the alerting network is dependent on the other two networks and cannot operate independently. Increased alertness modulates performances of the other attentional networks and facilitates their operation. A high alerting state improves RT in orienting and executive function tasks, and as a trade-off, results in an increase in error rates. In a state of high alertness, the selection of a response occurs more quickly, based upon a lower quality of information. This effect has a major influence on the executive function network in conflict situations. In the developmental dyscalculia group, we found that a high alerting state increased the congruity effect, more than in the controls. As mentioned earlier, alertness generally speeds up responding. However, because it increased the flanker effect in the developmental dyscalculia group much more than in the control group, the expected decrease in RT was not evident in incongruent trials.

### Deficit in orienting

We hypothesized that the developmental dyscalculia group would present deficits in the performance of the orienting network. The malfunction of the intraparietal sulcus in developmental dyscalculia participants was the basis of this prediction [16-18]. However, contrary to our hypothesis, no group difference was found in the orienting between the developmental dyscalculia participants and controls. Corbetta and Shulman [76] suggested that the intraparietal sulcus is more involved in endogenous orienting compared to exogenous orienting (but see appendix 2). The ANT-I paradigm examines the ability of the orienting network using exogenous attention. Hence, the present study revealed comparable exogenous orienting ability in developmental dyscalculia participants and controls. The possibility of difficulties in the endogenous orienting system in the developmental dyscalculia group remains open.

In relation to the interaction between executive function and orienting, developmental dyscalculia participants had a larger congruity effect in the non-cued condition. However, in the valid condition (i.e., line of arrows appeared in the cued location), they could filter irrelevant distractors or operate more control, similar to the control group. In the invalid trials, the two groups presented a larger congruity effect than in the valid trials. It seems that when stimuli appear in uncued locations, the two groups have less ability to operate control. In this case, the performance of the two groups appeared similar, and the advantage of the control group over the developmental dyscalculia group disappeared.

This pattern of results suggest that, similar to controls, a preparatory cue may improve the performance of the developmental dyscalculia group (i.e., reduce the congruity effect) in executive tasks. Moreover, this result reiterates the suggestion that those with developmental dyscalculia have a lower ability to monitor conflict, which can be reduced by focussing their attention on the spatial location of the stimuli.

### Developmental dyscalculia as non-unitary deficits

Is developmental dyscalculia a unique pathophysiology that involves abnormalities restricted to the intraparietal sulcus or is it due to multiple brain dysfunctions with multiple cognitive deficits [24]? The present study hints that those with developmental dyscalculia present multiple cognitive deficits. As mentioned earlier, Rotzer et al. [61] discovered abnormalities in the gray matter volume of developmental dyscalculia participants compared to controls, in frontal sites that are considered to be part of the executive functions network. In addition, neuroimaging studies discovered functional and structural abnormalities of the right intraparietal sulcus among those with developmental dyscalculia [e.g., [16,17,61]]. Hence, the cognitive deficits that might underlie developmental dyscalculia may be due to multiple brain abnormalities. It seems that the "pure" developmental dyscalculia we studied is characterized by multiple deficits that involve the executive function and alertness networks.

Finally, we do not claim that the deficit in attention is the only deficit observed in developmental dyscalculia or that the numerical deficiencies in developmental dyscalculia are due to attention deficits (as the domain-general hypothesis would suggest). We suggest that in developmental dyscalculia, numerical deficits are the crucial factor, but similar to other developmental disorder [e.g., attention deficits in dyslexia, see [78]], additional domains are also damaged but to a lower degree [25].

## Limitations

Conclusions based on the present findings are limited by several factors. Due to the rareness of pure developmental dyscalculia cases, we could only test a small sample of participants. Furthermore, the ANT-I task examines three attentional networks and their interaction in a single task. It is also important to examine the attentional abilities of developmental dyscalculia participants in every attentional network independently.

## Conclusions

The present study demonstrated deficits in the executive function and the alertness networks among those that were diagnosed as having pure developmental dyscalculia compared to matched controls. It seems that participants that were diagnosed as having pure developmental dyscalculia (with no indication of ADHD) presented attention deficits that were not directly related to numerical processing and that were different from the ones usually observed in those ADHD. It seems that developmental dyscalculia involves certain aspects that are domain-general.

## Appendices

### Appendix 1

There are several differences between the two tasks. 1) The ANT and the ANT-I both examine the executive function network using flankers with congruent and incongruent conditions. The ANT uses neutral trials whereas the ANT-I does not. 2) The orienting system is tested using an asterisk above or below the fixation point. The ANT uses only a valid/predictive (endogenous) cue. Namely, there are two conditions: cued, where the target appears at the cued location (100% predictive), or uncued, where the target follows a central cue. The ANT-I uses a nonpredictive cue (exogenous) with valid (target at cued location), non-cued (no spatial cue is presented), and invalid (target appears opposite to the cued location) conditions. 3) The alertness of attention is manipulated differently in the two tasks. In the ANT it is examined using two asterisks (double cue), while in the ANT-I a high pitched tone is used.

### Appendix 2

Note however, that others have distinguished between a posterior and an anterior system of attention; (a) an automatic posterior attention system involves the superior parietal cortex (intraparietal sulcus); and (b) a voluntary anterior attention system involves frontal sites. According to this view, the intraparietal sulcus is more related to exogenous attention [e.g., [77]]. This would lead one to expect a difference in exogenous attention between those with developmental dyscalculia and controls, which was not found.

## Competing interests

The authors declare that they have no competing interests.

## Authors' contributions

AS contributed in developing the study paradigm, recruited subjects, conducted the testing, diagnosed the developmental dyscalculia group and the control group, had a major role in data interpretation and prepared the manuscript. AH played a major role in developing the study design, interpretation and analysis of the data, and contributed to scientific writing, in addition to critically reviewing the manuscript. All authors read and approved the final manuscript.
